# Histomorphometric changes of small intestine in pregnant rat

**Published:** 2015-03-15

**Authors:** Fatemeh Sabet Sarvestani, Farhad Rahmanifar, Amin Tamadon

**Affiliations:** 1 *Graduated from School of Veterinary Medicine, Shiraz University, Shiraz, Iran; *; 2 * Department of Basic Sciences, School of Veterinary Medicine, Shiraz University, Shiraz, Iran; *; 3 *Transgenic Technology Research Center, Shiraz University of Medical Sciences, Shiraz, Iran.*

**Keywords:** Histomorphometry, Pregnancy, Rat, Small intestine

## Abstract

Food intake of rats increases during pregnancy. This requires changes in the structure of the small intestine to absorb additional food. The aim of the present study was to investigate the morphological changes in the layers of small intestine in rats during pregnancy. Duodenum, jejunum and ileum of 18 pregnant Sprague-Dawley rats (day 7, 14 and 21 of pregnancy) were collected. Villous height and width and thickness of lamina propria, tunica muscularis entirely and separately (circular and longitudinal layers) were measured on transverse sections. During pregnancy increasing villi length and muscular layer thickness was observed in duodenum. Furthermore, along with the progress of gestation greatest histomorphometric change in small intestine was observed in the jejunum. The reduction in the ileum histomorphologic indices was observed during pregnancy. In conclusion, increase in histomorphologic indices of duodenum and jejunum supplies more capacity of duodenum to digest food intake during pregnancy and decrease in these indices in ileum controls the absorption of excess produced amino acids and glucose by hyperphagia.

## Introduction

The intestinal tract is a hollow muscular tube serving as a digestive organ. The small intestine transports, digests and absorbs nutrients and comprises the duodenum, jejunum and ileum.^1^^,^^2^ Similar to the other organs, the intestine undergoes a pattern of adaptive changes during postnatal development. In the intestinal mucosa, such as villi, crypts, enterocytes and microvilli these changes occur largely in structures related to the exchange and absorption processes. The morphological and biochemical changes that occur in the human intestinal mucosa under different physiological and pathological conditions have been studied in rat as an animal model.^3^^-^^5^ 

Feed intake increases by 40 to 50% during pregnancy in the rat.^   6 ^ In the lactating rat, food consumption is 2.5 to 3.0 times greater than that of non-lactating, non-pregnant animals.^7^^,^^8^ This increased consumption was found to return to pre-copulation levels within day 9 of weaning.^   7 ^ During lactation, the rat alimentary canal increases in weight, length and nitrogen content.^8^^-^^12^ Hypertrophy of all layers of the small intestinal wall has been reported in the late stages of pregnancy and during lactation in rats.^8^^,^^13^ However, Boyne *et al*. reported an increase in intestinal mucosal surface area during lactation.^   13 ^ There are a number of reports demonstrating hormonal modification of intestinal absorption.^14^^-^^16^ Although little is known about the effect of pregnancy on this function. To this aim, in this study, the morphological changes in the villi, lamina propria, and tunica muscularis of small intestine were investigated in rat during pregnancy.

## Materials and Methods

**Fig. 1:**
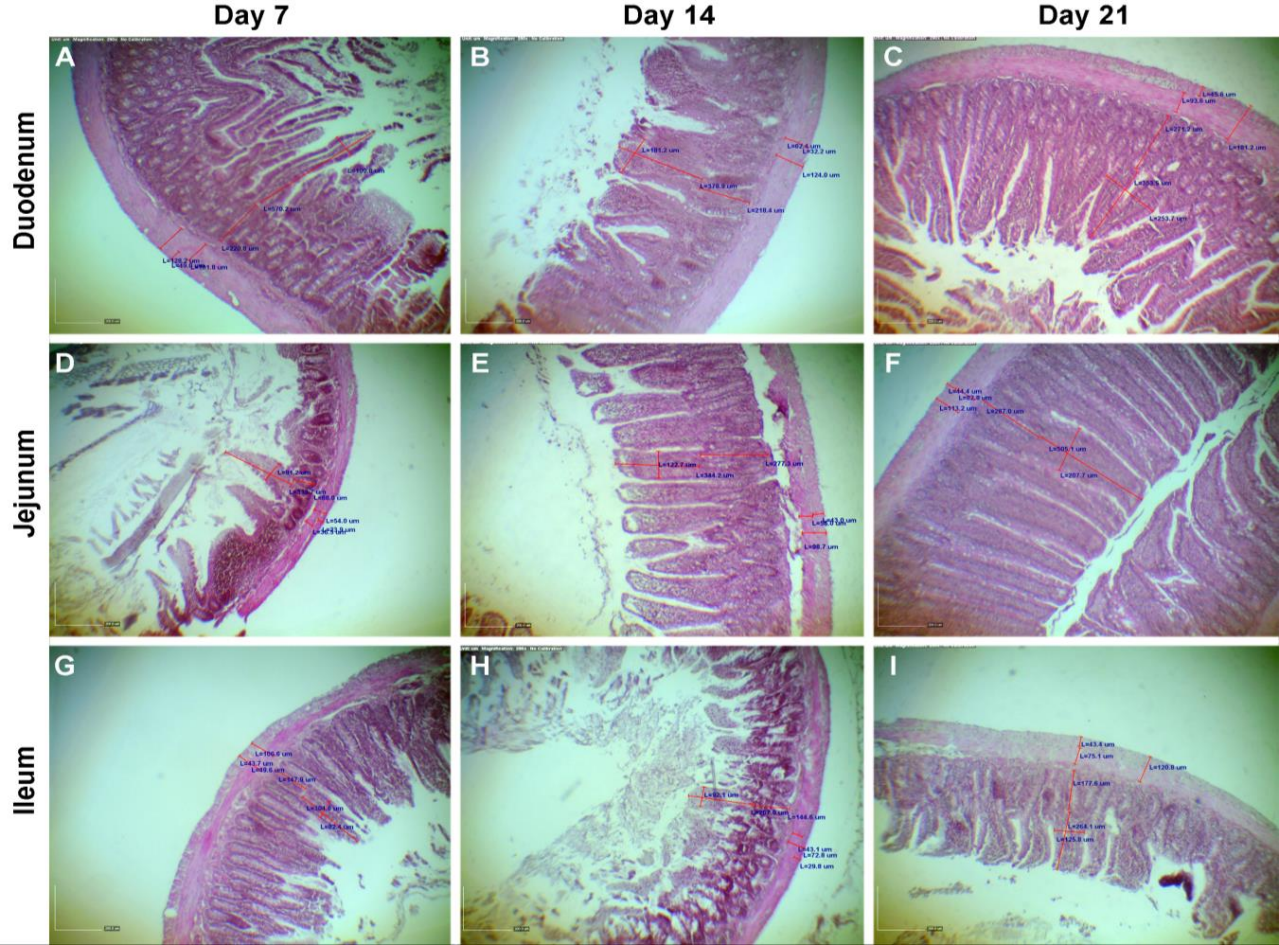
Histomorphometry of small intestine at different ages of pregnancy in female rats. Length and width of villi, diameter of lamina propria, tunica muscularis, and the first and the second layer of tunica muscularis were measured (H&E, 290×).

Eighteen adult (3 to 4 months old) female Sprague-Dawley rats (*Rattus norvegicus*) weighing between 170 to 220 g were used in this randomized controlled study. The rats were housed in standard cages, three per cage, in a controlled temperature room (22 ˚C), with a 12 hr light and 12 hr dark cycle. Standard laboratory chow and tap water were available *ad libitum*. The female rats in estrus or pro-estrus (using vaginal smear) were mated with the male rat (3:1) and the exact pregnancy day of the rats was confirmed using the vaginal smear method.^   17 ^ They were randomly assigned in three equal groups of 7, 14, and 21 days of pregnancy (n = 6) and the rats were killed ethically. At first, their pregnancy was confirmed certainly by observation of their pregnant uterus. The segments of the duodenum (2 cm posterior to stomach), jejunum (2 cm anterior to Meckel´s diverticulum) and ileum (2 cm anterior to ileocaecal junction) were collected. Samples of the intestinal segments were fixed in 10% buffered formalin solution. Serial transverse sections of 5 μm thickness were cut along the intestine fragments. All sections were stained with hematoxylin and eosin. Sections were visualized and photographed on light microscope (Model CX21; Olympus, Tokyo, Japan) equipped with an adjusted digital camera (Model AM423U Dino-Eye; Dino-Lite, San-Chung, Taiwan). Villous height and width, thickness of tunica submucosa, tunica muscularis entirely and separately (circular and longitudinal layers) were measured on transverse sections using Dino Capture software (Version 2.0; Dino-Lite, San-Chung, Taiwan), (Fig. 1).

The mean values ± standard error (SE) of small intestine histomorphometry were subjected to Kolmogorov-Smirnov test of normality and analyzed by one-way ANOVA (Version 11.5; SPSS Inc., Chicago, USA), and post-hoc test was performed by Tukey's test. A *p*-value less than 0.05 was considered to be statistically significant. Group means and their standard error are reported in the text and graphs using GraphPad Prism (Version 5.01; GraphPad software Inc., San Diego, USA).

## Results

**Fig. 2:**
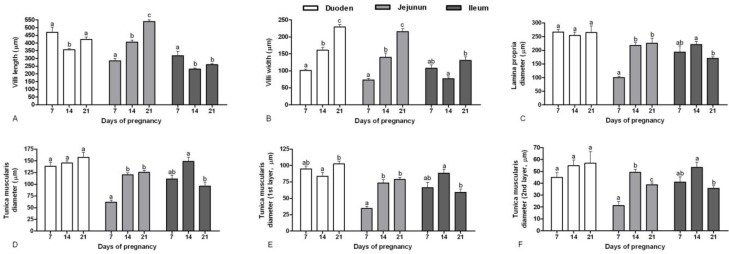
Histomorphometry results (Mean ± standard error) of small intestine at different ages of pregnancy in female rats. A, villi length (μm); B, villi width (μm); C, lamina propria diameter (μm); D, tunica muscularis diameter (μm); E, the first layer of tunica muscularis diameter (μm); and F, the second layer of tunica muscularis diameter (μm).

^abc^ different letters show statistically significant differences between days of pregnancy in the same part of small intestine (*p* < 0.05).

Villi length in duodenum was decreased until day 14 of pregnancy (*p* = 0.001) and then increased near parturition, on day 21 (*p* = 0.04, Fig. 2A). In the jejunum this item was increased during growth of fetuses (*p* < 0.001). In the ileum, the length of villi was decreased with age until day 14 of pregnancy (*p* < 0.001) and did not increase until parturition, on day 21 (*p* = 0.15). Villi width in duodenum and jejunum was increased during pregnancy (*p* < 0.001), but in ileum decreased until day 14 (*p* = 0.06) and then increased on day 21 (*p* < 0.001, Fig. 2B).

Lamina propria diameter in duodenum did not have any remarkable changes during pregnancy (*p* > 0.05, Fig. 2C). However, in jejunum, it was increased until day 14 pregnancy (*p* < 0.001) and remained unchanged until the end of pregnancy. Most diameter of lamina propria was on day 14 of pregnancy in ileum and then decreased until day 21 of pregnancy (*p* = 0.007).

Tunica muscularis diameter in duodenum did not have any significant growth during pregnancy (*p* > 0.05, Fig. 2D). However, in jejunum it was increased until day 14 pregnancy (*p* < 0.001) and remained unchanged until the end of pregnancy. Most diameter of tunica muscularis was on day 14 of pregnancy in ileum and then decreased until day 21 of pregnancy (*p* < 0.001). The diameter of the first layer of tunica muscularis in duodenum was remained unchanged until day 14 (*p* = 0.31) and then increased on day 21 (*p* = 0.03, Fig. 2E). However, in jejunum it was increased until day 14 of pregnancy (*p* < 0.001) and remained unchanged until the end of pregnancy (*p* = 0.61). Most diameter of the first layer of tunica muscularis was on day 14 of pregnancy in ileum and then decreased until day 21 of pregnancy (*p* = 0.003). The diameter of the second layer of tunica muscularis in duodenum did not have any significant change during pregnancy (*p* > 0.05, Fig. 2F).

The diameter of the second layer of tunica muscularis in jejunum was increased until day 14 of pregnancy (*p* < 0.001) and then decreased until day 21 of pregnancy (*p* = 0.03).

Most diameter of the second layer of tunica muscularis was on day 14 of pregnancy in ileum and then decreased until day 21 (*p* = 0.008).

## Discussion

There is no increase in energy efficiency during gestation and the energy balance becomes positive at this stage primarily because of an increase in food intake which is necessary to prevent the depletion of maternal energy stores.^   18 ^ Results of the present study showed that villi width, tunica muscularis, and the second layer of tunica muscularis diameter of duodenum were increased during pregnancy in rat. Consistent with our findings, Prieto *et al*. showed that pregnancy induces hypertrophy of duodenum in rat.^   19 ^ Moreover, in jejunum of rat, villous height and width, lamina propria, and tunica muscularis thickness were increased during pregnancy which these findings were similar to findings of Prieto *et al*. in pregnant rat jejunum.^   19 ^ Nevertheless, in this study in ileum of pregnant rat, just villi width was increased after day 14 of pregnancy, however, other indices were decreased with pregnancy progress which was contrary to Prieto *et al*. findings which reported hypertrophy of ileum in pregnant rat.^   19 ^ Moreover, increases in small intestinal mucosal area, elongation and thickening of villi and greater numbers of enterocytes in late pregnancy and particularly during lactation have been reported in rat.^13,^^20^^,^^21^ Dunn reported that during the first stage of development, day 13 to 18 of pregnancy, rapid expansion of the epithelium linked with the development of the muscle wall was occurred in pregnant rat.^   22 ^ On day 18, localized areas of epithelial proliferation appear and were soon invaded by connective tissue to form early villi.^   22 ^ From day 18 to birth cellular differentiation was prominent including the growth and proliferation of microvilli, the development of the mitochondria, the replacement of ribosomal polysomes by organized rough endoplasmic reticulum, and the shrinkage of the nucleus.^   22 ^ Together with the previous findings our data confirmed that histomorphometric alterations during pregnancy in rat were not the same in different parts of small intestine.

The duodenum is responsible for the enzymatic breakdown of food, therefore, it seems increase of villi width and tunica muscularis which observed in the present study by progressing of pregnancy can be reasonable in rat. During pregnancy of rat, food intake increases by more than 50%,^   20 ^ therefore, duodenum should gain new abilities to increase the digestibility of the extra volume of food. The rat can cope with digestion and absorption of a 60% increase in food intake with little adjustment of the gut.^   20 ^ The epithelial layer of the jejunum is specialized for the absorption of most of nutrient particles. Moreover, the length of the small intestine of pregnant rats was the same as that in virgins. There was a significant increase in total wet and dry weight and in wet and dry weight per cm length.^   21 ^ For this reasons, with increasing gestational age, thickening of the entire indices of jejunum was observed to absorb the additional food.

The large numbers of capillaries in the villi of ileum are responsible for the absorption of amino acids and glucose produced by digestion. Absolute absorption of leucine and glucose is not significantly enhanced during pregnancy of rat.^   20 ^ Therefore, decrease of ileum indices which were observed in the present study can be reasonable in rat.

On the other hand pregnancy is accompanied by numerous changes in hormone secretion that participate in the adaptation of the mother to the gestational condition. Pregnancy is a physiological state characterized by the presence of hyperprolactinemia. Several data appear to show that prolactin may be a major factor mediating the hyperphagia associated with both states.^   23 ^ In fact, there are reports indicating that prolactin can induce hyperphagia when administered systemically or intra-cerebro-ventricularly. ^24^^,^^25^  The hyperprolactinemia exhibited in both states likely plays an important role in regulating numerous brain functions, including feeding and appetite.^   23 ^ During the first three days of pregnancy, serum prolactin was higher than during the following day 18. On day 22 of pregnancy serum prolactin levels rose to three times more than day 18.^   26 ^

In conclusion, more capacity of duodenum to digest food intake during pregnancy was observed by increasing villi length and muscular layer thickness. Furthermore, along with the progress of gestation, the greatest histo-morphometric change in small intestine was observed in the jejunum to increase absorption of nutritional needs during pregnancy with progression of pregnancy in rat. The reduction in the ileum histomorphologic indices during pregnancy control the absorption of excess produced amino acids and glucose by hyperphagia.
